# Reversion of methionine addiction of osteosarcoma cells to methionine independence results in loss of malignancy, modulation of the epithelial-mesenchymal phenotype and alteration of histone-H3 lysine-methylation

**DOI:** 10.3389/fonc.2022.1009548

**Published:** 2022-11-03

**Authors:** Yusuke Aoki, Qinghong Han, Yasunori Tome, Jun Yamamoto, Yutaro Kubota, Noriyuki Masaki, Koya Obara, Kazuyuki Hamada, Justin D. Wang, Sachiko Inubushi, Michael Bouvet, Steven G. Clarke, Kotaro Nishida, Robert M. Hoffman

**Affiliations:** ^1^ AntiCancer Inc, San Diego, CA, United States; ^2^ Department of Surgery, University of California San Diego, La Jolla, CA, United States; ^3^ Department of Orthopedic Surgery, Graduate School of Medicine, University of the Ryukyus, Nishihara, Japan; ^4^ School of Medicine, California University of Science and Medicine, Colton, CA, United States; ^5^ Department of Surgery, Kobe University, Kobe, Japan; ^6^ Department of Chemistry and Biochemistry, University of California Los Angeles, Los Angeles, CA, United States

**Keywords:** malignancy, methionine addiction, Hoffman effect, methionine-independent revertant, epithelial-mesenchymal phenotype, histone-methylation, osteosarcoma

## Abstract

Methionine addiction, a fundamental and general hallmark of cancer, known as the Hoffman Effect, is due to altered use of methionine for increased and aberrant transmethylation reactions. However, the linkage of methionine addiction and malignancy of cancer cells is incompletely understood. An isogenic pair of methionine-addicted parental osteosarcoma cells and their rare methionine-independent revertant cells enabled us to compare them for malignancy, their epithelial-mesenchymal phenotype, and pattern of histone-H3 lysine-methylation. Methionine-independent revertant 143B osteosarcoma cells (143B-R) were selected from methionine-addicted parental cells (143B-P) by their chronic growth in low-methionine culture medium for 4 passages, which was depleted of methionine by recombinant methioninase (rMETase). Cell-migration capacity was compared with a wound-healing assay and invasion capability was compared with a transwell assay in 143B-P and 143B-R cells *in vitro*. Tumor growth and metastatic potential were compared after orthotopic cell-injection into the tibia bone of nude mice *in vivo*. Epithelial-mesenchymal phenotypic expression and the status of H3 lysine-methylation were determined with western immunoblotting. 143B-P cells had an IC_50_ of 0.20 U/ml and 143B-R cells had an IC_50_ of 0.68 U/ml for treatment with rMETase, demonstrating that 143B-R cells had regained the ability to grow in low methionine conditions. 143B-R cells had reduced cell migration and invasion capability *in vitro*, formed much smaller tumors than 143B-P cells and lost metastatic potential *in vivo*, indicating loss of malignancy in 143B-R cells. 143B-R cells showed gain of the epithelial marker, ZO-1 and loss of mesenchymal markers, vimentin, Snail, and Slug and, an increase of histone H3K9me3 and H3K27me3 methylation and a decrease of H3K4me3, H3K36me3, and H3K79me3 methylation, along with their loss of malignancy. These results suggest that shifting the balance in histone methylases might be a way to decrease the malignant potential of cells. The present results demonstrate the rationale to target methionine addiction for improved sarcoma therapy.

## Introduction

Cancers were originally described as methionine dependent, as it was thought that the cancer cells lost the ability to synthesize methionine (1–3). However, we showed that cancer cells make normal or greater-than-normal amounts of methionine from homocysteine, but still require large amounts of exogenous methionine in order to grow and survive, unlike normal cells, due to excess transmethylation reactions (4–10). Our seminal studies of methionine addiction of cancer cells (4–9) were confirmed by Wang, et al., who showed that tumor-initiating cells were highly methionine-addicted (11). We further characterized methionine addiction of cancer cells (12–16). Thus, cancer cells must have much larger-than-normal amounts of methionine in order to survive and proliferate, which is described as methionine addiction. Other teams are now using the term “methionine addiction of cancer” (11, 17, 18). We have previously shown that osteosarcoma cells, including 143B, Saos-2, MNNG-HOS, and U-2OS, are methionine-addicted, and 143B is the most methionine addicted among these osteosarcoma cell lines (19). However, the linkage of the methionine addiction and malignancy is incompletely understood.

Methionine-independent revertant cells, isolated from methionine-addicted parental cancer cells in low-exogenous methionine conditions (20), have reduced malignancy (13, 15, 16, 21, 22), suggesting that reversion of methionine addiction of cancer cells to methionine independence is related to malignancy itself. We and Borrego et al. have previously shown that methionine-independent revertant cells have reduced growth ability in soft agar (16, 21, 22). We have also shown methionine-independent revertant cells have lost tumorgenicity in subcutaneous-xenograft mouse models (13, 16) and lost metastasis in xenograft mouse models (15).

It was previously reported that methylation of histone H3 lysine marks in tumor-initiating cells was increased compared to non-tumor-initiating cells (11). Our previous studies also showed that histone-H3 lysine-methylation was increased in cancer cells compared to normal cells (12, 23), and it is further increased in high-malignancy variants selected from parental cancer cells, compared to the parental cells (16). We also previously reported that the status of the histone-H3 lysine-methylation in methionine-addicted cancer cells is unstable during methionine restriction, using recombinant L-methionine α-deamino-γ-mercapto-methane lyase (rMETase) (24), at concentrations which arrest cancer-cell proliferation (12, 14). These results suggest that the status of the histone-H3 lysine-methylation might be associated with malignancy ofcancer cells. However, the molecular relationship of methionine addiction and malignancy is incompletely understood.

In osteosarcoma, the 5-year survival rate for patients who have metastases, mostly lung metastasis, is about 30%, while the 5-year survival rate for overall osteosarcoma patients is about 60% (25). In the metastatic processes, epithelial-to-mesenchymal transition (EMT) and mesenchymal-to-epithelial transition (MET) are thought to be required for cell plasticity (26). Recently, EMT/MET has been studied in osteosarcoma (27–37) and other types of sarcoma (38–45), as well as carcinomas, since the theory that sarcoma cells can reside in an intermediate phenotype between epithelial and mesenchymal has been widely accepted (46–48).

In the present report, we show that reversion of methionine addiction of osteosarcoma cells to methionine-independence results in loss of malignancy, modulation of the epithelial-mesenchymal phenotype, and alteration of histone-H3 lysine-methylation. The present results provide unique clues for further understanding of the fundamental basis of cancer.

## Materials and methods

### Cell culture

The 143B human osteosarcoma cell line was obtained from the American Type Culture Collection (Manassas, VA, USA) and cultured in Dulbecco’s Modified Eagle Medium (DMEM; #10-103-CV, Corning Inc., Corning, NY, USA), supplemented with 1 IU/ml penicillin/streptomycin (#15-240-062, Thermo Fisher Scientific, Waltham, MA, USA) and 10% fetal bovine serum (FBS; Access Biologicals, Vista, CA, USA).

### Recombinant methioninase production

Recombinant methioninase (rMETase) is a tetramer, with each monomer having a 172-kDa molecular weight. The procedure for the production of rMETase, from recombinant E. coli with the Pseudomonas putida gene, has been previously reported (24).

### Depletion of methionine in the culture medium with rMETase

Normal methionine-containing DMEM with/without different amount of rMETase, 0 U/ml; 0.125 U/ml; 0.25 U/ml; 0.5 U/ml; 1 U/ml; 2 U/ml; and 4 U/ml, was incubated at 37°C for 3 h. The concentration of methionine in each medium was determined with an HPLC (Hitachi L-6200A Intelligent pump, Hitachi, Ltd., Tokyo, Japan). Experiments were performed twice.

### Selection for methionine-independent revertant osteosarcoma cells

Methionine-independent revertant 143B osteosarcoma cells (143B-R) were selected by the following procedure, modified from previous reports (13, 15, 16): methionine-addicted parental 143B osteosarcoma cells (143B-P) were cultured in normal medium with increasing concentrations of rMETase (0.75-1.6 U/ml) for 3 weeks, followed by culture in normal medium for 3 weeks and passage. This procedure was repeated 4 times.

### rMETase sensitivity assay

Each 143B-P and 143B-R cells were cultured in 96-well plates (1.0 × 10^3^ cells/well) in normal DMEM (100 μl/well) and incubated at 37°C overnight. Each cell was treated with normal medium or different concentration of rMETase, at 37°C for 72 h, as follows: 0 U/ml; 0.025 U/ml; 0.05 U/ml; 0.1 U/ml; 0.2 U/ml; 0.4 U/ml; 0.8 U/ml; and 1.6 U/ml. After the treatment period, WST-8 reagent (10 μl) (#CK04, Dojindo laboratory, Kumamoto, Japan) was added to each well and the plate was additionally incubated at 37°C for 1 h. Absorbance at 450 nm was measured, and rMETase-sensitivity cell-survival curves and half-maximal inhibitory concentration (IC_50_) were obtained, as previously described (14). Experiments were performed twice in triplicate.

### Wound healing assay

Each 143B-P and 143B-R cells were cultured in 6-well plates in normal medium and incubated at 37°C overnight. Wounds were made by scratching the monolayers with a 200 μl pipette tip. The plates were washed with phosphate-buffered saline (PBS; #MB1039-1X, BioPioneer Inc., San Diego, CA, USA) twice to remove floating cells, followed by incubation at 37°C. The wound areas were measured by light microscopy (Olympus IX70, Olympus Corporation, Tokyo, Japan) at four and eight hours after making the wound. Experiments were performed twice in triplicate.

### Invasion assay

24-well Transwell inserts (#3422, Corning Inc.) were coated with 100 μl of Matrigel Matrix (200 μg/ml) (#354234, Corning Inc.) for each well, in advance. 143B-P or 143B-R cells were cultured in the upper chambers of the insert (2.5 × 10^4^ cells/well) with 250 μl FBS-free medium, with the lower chambers filled with normal medium, containing 10% FBS, followed by incubation at 37°C for 18 h. The inserts were removed and washed with PBS twice, followed by fixation with 100% methanol (#33900HPLC, Pharmco, Brookfield, CT, USA) for 20 min and staining with 0.5% crystal violet (#C0775-25G, Sigma-Aldrich, Burlington, MA, USA) for 15 min. The inner side of the inserts was wiped with cotton swabs. The remaining cells were counted under a light microscopy (Olympus IX70, Olympus Corporation). Experiments were performed twice in triplicate.

### Western immunoblotting

143B-P or 143B-R cells were cultured in 25 cm^2^ cell-culture flasks in normal DMEM for more than 5 days. Cells were then lysed to extract total proteins, using RIPA Lysis and Extraction buffer (#89900, Thermo Fisher Scientific) with 1% Halt Protease Inhibitor Cocktail (#87786, Thermo Fisher Scientific). Extraction of total histones used an Epiquik Total Histone Extraction Kit (#OP-0006-100, Epigentek, Farmingdale, NY, USA). Western immunoblotting for total proteins and histones was performed as follows: Total proteins or histones were loaded onto 7.5% or 12% SDS-PAGE gels, for electrophoresis, respectively. After separation of proteins or histones by electrophoresis, they were transferred to 0.45 μm or 0.2 μm polyvinylidene difluoride (PVDF) membranes (#GE10600023 or #GE10600021, respectively, GE Healthcare, Chicago, IL, USA). The membranes were blocked with Bullet Blocking One for Western Blotting (#13779, Nakalai Tesque, Inc., Kyoto, Japan). Anti-ZO-1 antibody (1:1,000, #8193, Cell Signaling Technology, Danvers, MA, USA); anti-vimentin antibody (1:1,000, #5741, Cell Signaling Technology); anti-Snail antibody (1:1,000, #3879, Cell Signaling Technology); anti-Slug antibody (1:1,000, #9585, Cell Signaling Technology); anti-beta actin antibody (1:1,500, #20536-1-AP, Proteintech, Rosemont, IL, USA); anti-H3K4me3 antibody (1:1,000, #9751, Cell Signaling Technology); anti-H3K9me3 antibody (1:1,000, #13969, Cell Signaling Technology); anti-H3K27me3 antibody (1:1,000 #9733, Cell Signaling Technology); anti-H3K36me3 antibody (1:1,000, #4909, Cell Signaling Technology); anti-H3K79me3 antibody (1:1,000, #74073, Cell Signaling Technology); anti-H3 antibody (1:1,500, #17168-1-AP, Proteintech) were used. Beta-actin or total histone-H3 were used as internal loading controls. Horseradish-peroxidase-conjugated anti-rabbit IgG (1:20,000, #SA00001-2, Proteintech) was used as the secondary antibody. The signals were detected with a UVP ChemStudio (Analytik Jena, Upland, CA, USA), enhanced by Clarity Western ECL Substrate (#1705061, Bio-Rad Laboratories, Hercules, CA, USA). The protein signals were normalized to those of beta-actin, the histone signals were normalized to those of total histone-H3, for relative quantification. Experiments were performed three times.

### Animals

Female athymic nu/nu nude mice (4-6-week-old) (AntiCancer Inc., San Diego, CA, USA) were used for the present study. All mice were bred and maintained in a barrier facility with high efficacy particulate air (HEPA) -filtered rack under standard conditions (12 h light/dark cycles). The care and use of animals was reviewed and approved under Assurance Number A3873-1, with The Institutional Animal Care and Use Committee (IACUC) protocol, based on the National Institutes of Health (NIH) Guide for the Care and Use of Animals, approved. A cocktail of anesthetics and analgesics [ketamine (20 mg/kg) (#11695-0702-1, Henry Schein, Inc., Melville, NY, USA), xylazine (15.2 mg/kg) (#59399-111-50, Akorn Operating Company LLC, Lake Forest, IL, USA), acepromazine maleate (0.48 mg/kg) (#0010-3827-01, Boehringer Ingelheim GmbH, Ingelheim, Germany)] was used for all surgical procedures to minimize animal distress.

### Osteosarcoma orthotopic xenograft mouse model

143B-P or 143B-R cells [2.5 × 10^5^ cells/5 μl PBS and 5 μl Matrigel Matrix (#354234, Corning Inc.)] were injected with 28G syringes (#329461, BD, Franklin Lakes, NJ, USA) into the left proximal tibia of ten mice each. The procedure was performed as described in previous reports (49, 50). Tumor size was measured twice a week with calipers and calculated with the following formula: tumor volume (mm^3^) = length (mm) × width (mm) × width (mm) × 1/2. All mice were sacrificed four weeks after cell injection. Tumor and lung samples were obtained to examine spontaneous metastases in the lungs macroscopically and with subsequent hematoxylin and eosin (H&E) staining.

### H&E staining

From each mouse with a primary tumor in the tibia at necropsy, tumor tissue and lung tissues were obtained and fixed with 10% formalin (#SF99-20, Thermo Fisher Scientific) for 48 h, followed by embedding in paraffin (#PARA2, Cancer Diagnostics, Inc., Durham, NC, USA). The tissues were then sectioned, deparaffined, and rehydrated. H&E staining was performed according to standard protocols.

### Statistical analysis

The Student’s t-test was performed to statistically analyze the means between two groups, and the Fisher’s exact test was performed to statistically evaluate the metastatic potential of 143B-P and 143B-R cells, with JMP pro ver. 15.0.0 (SAS Institute, Cary, NC, USA). IC_50_ values were obtained with ImageJ ver. 1.53a (National Institutes of Health, Bethesda, MD, USA). Bar graphs show the mean and error bars show standard deviation of the mean. A probability value ≤ 0.05 was defined as a statistically-significant difference.

## Results

### Methionine-independent revertant osteosarcoma cells were selected from methionine-addicted parental osteosarcoma cells in low-methionine medium

**Figure 1:**
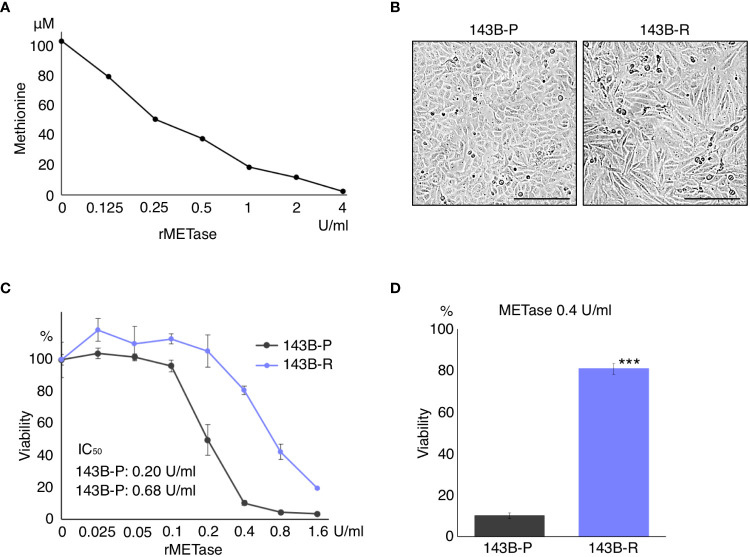
Methionine-independent revertant osteosarcoma cells become less-sensitive to methionine restriction. **(A)** Dose-dependent methionine depletion of DMEM effected by rMETase. The methionine level in DMEM was measured 3 h after rMETase was added. **(B)** Morphology of 143B-P and 143B-R cells. Scale bar in photomicrographs: 250 μm. Magnification: 100×. **(C, D)** Methionine-restriction sensitivity of 143B-P and 143B-R cells with rMETase. 143B-P: methionine-addicted parental 143B osteosarcoma cells, 143B-R: methionine-independent 143B osteosarcoma cells. ***P < 0.001.

rMETase decreased the level of methionine in the culture medium, in a concentration-dependent manner (**Figure 1A**). 143B-R cells were selected from 143B-P cells in low methionine media after 4 cycles of selection. 143B-R cells were more normal-fibroblast-like, becoming larger and more bipolar and aligned in direction than 143B-P cells, which is consistent with previous reports of other methionine-independent revertant cells (21) (**Figure 1B**). 143B-R cells became resistant to methionine restriction, induced by rMETase, compared to 143B-P, with the following IC_50_: 143B-P: 0.20 U/ml; 143B-R: 0.68 U/ml (P < 0.001), which is consistent with previous reports of other methionine-independent revertant cells from various cancer types (15, 20, 22, 51) (**Figures 1C, D**).

### Methionine-independent revertant osteosarcoma cells had reduced cell migration and invasion capacity

**Figure 2:**
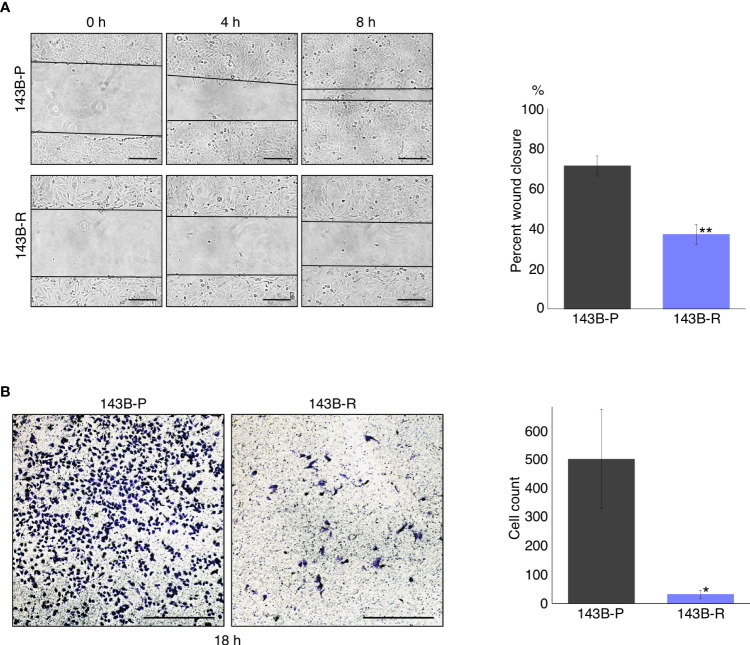
Methionine-independent revertant osteosarcoma cells have reduced cell migration and invasion capacity. **(A)** Cell migration capacity of 143B-P and 143B-R cells, in the wound-healing assay. Scale bar in photomicrographs: 250 μm. Magnification: 100×. **(B)** Cell-invasion capacity of 143B-P and 143B-R cells, with the transwell assay. Scale bar in photomicrographs: 250 μm. Magnification: 100×. 143B-P: methionine-addicted parental 143B osteosarcoma cells, 143B-R: methionine-independent 143B osteosarcoma cells. *P < 0.05, **P < 0.01.

A wound healing assay and cell-invasion assay were performed to compare the cell migration and invasion capacity of 143B-R cells and 143B-P cells. 143B-R cells showed significantly decreased cell migration (P < 0.01) and invasion (P = 0.018) capacity, compared to 143B-P (**Figure 2**), indicating that methionine-independent 143B-R cells lost malignancy *in vitro*.

### Methionine-independent revertant osteosarcoma cells had reduced tumor growth and lose metastatic potential *in vivo*

**Figure 3:**
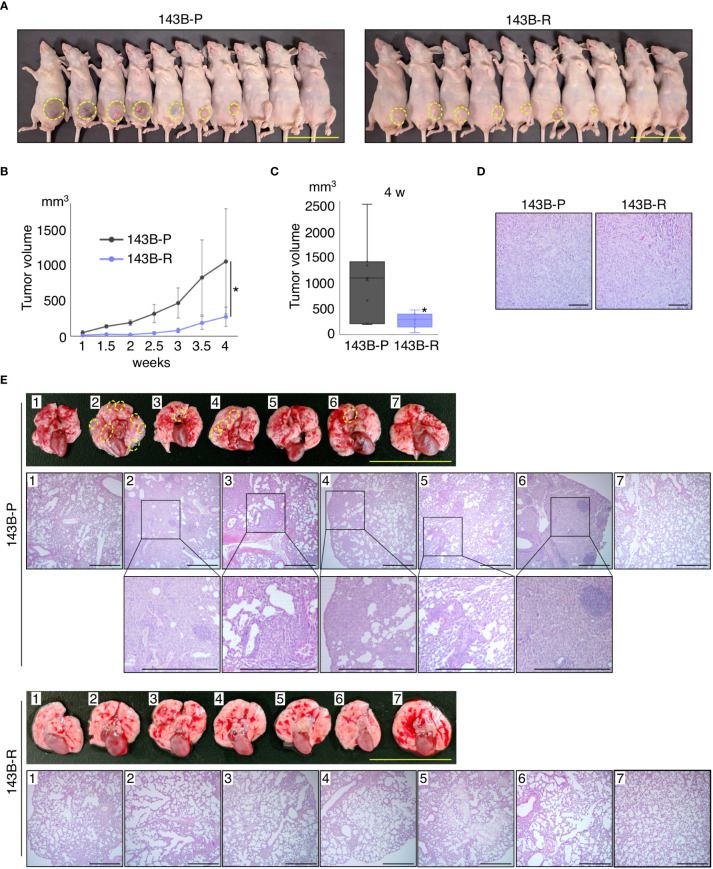
Methionine-independent revertant osteosarcoma cells have reduced tumor growth and lost metastatic potential, in orthotopic xenograft mouse models. **(A–C)** Tumor growth in the orthotopic xenograft mouse models of 143B-P or 143B-R cells. Dashed yellow lines show the edge of the tumor tissue. Scale bar: 50 mm. *P < 0.05. **(D)** Representative photomicrographs of H&E-stained primary tumor tissues in the tibia of 143B-P and 143B-R cells. Scale bar: 100 μm. Magnification: 100×. **(E)** Spontaneous lung metastases from the tibia of 143B-P and 143B-R cells. Dashed yellow lines show metastatic lesions in the lung. Scale bar in photographs: 25 mm. Scale bar in photomicrographs: 500 μm. Magnification of photomicrographs: 40×. 143B-P: methionine-addicted parental 143B osteosarcoma cells, 143B-R: methionine-independent 143B osteosarcoma cells.

To compare the metastatic potential of 143B-P and 143B-R cells *in vivo*, orthotopic xenograft mouse models, in which 143B-P or 143B-R cells were injected in the proximal tibia, were established and spontaneous lung metastasis from the tibia was examined. The 143B-R tumor size was significantly smaller than the 143B-P tumor size (P = 0.034), although both of them formed primary tumor tissue at the same ratio, 7 out of 10 mice (**Figures 3A–C**). Histological differences of the primary tumor tissues in 143B-P and 143B-R were not demonstrated with H&E staining (**Figure 3D**). Four different lung samples were then randomly obtained from each of the 7 mice with primary tumor. The lungs were examined for metastases from the primary tibia tumor. Macroscopically, 4 out of 7 lungs in 143B-P-injected mice had metastatic lesions, in contrast, no macroscopic metastatic lesions were seen in 143B-R mice (**Figure 3E**). Histological analysis demonstrated that 143B-P cells formed spontaneous lung metastases in 5 out of 7 mice, in contrast, 143B-R cells formed no metastases (P = 0.021) (**Figure 3E**). These results indicate that methionine-independent 143B-R cells lost malignancy *in vivo*, as well as *in vitro*.

### Expression of an epithelial marker was increased and the expression of mesenchymal markers were decreased in methionine-independent revertant osteosarcoma cells

**Figure 4:**
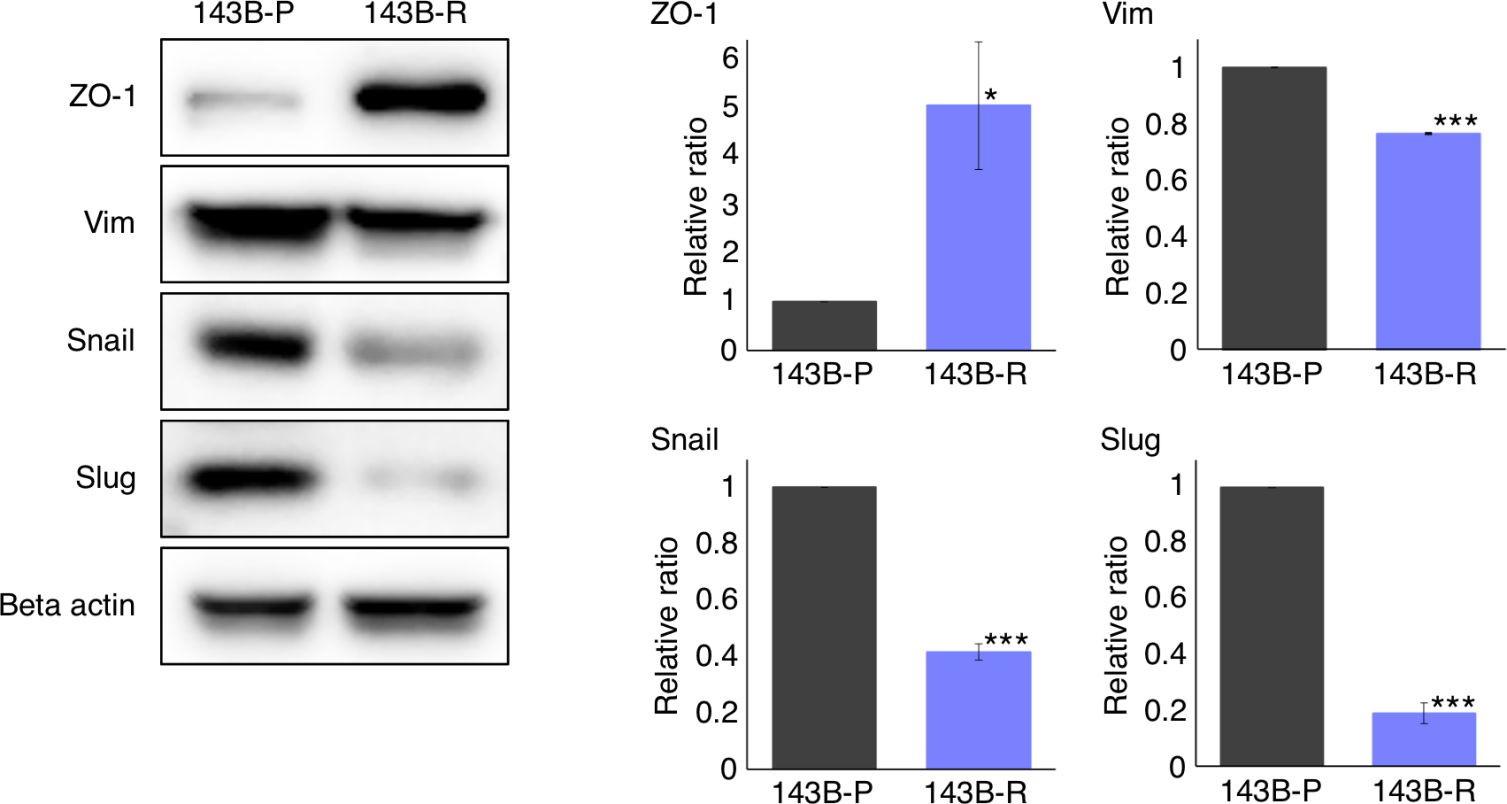
The expression of epithelial and mesenchymal markers in 143B-P and 143B-R cells, demonstrated with western immunoblotting. 143B-P: methionine-addicted parental 143B osteosarcoma cells, 143B-R: methionine-independent 143B osteosarcoma cells. *P < 0.05, ***P < 0.001.

We then performed western immunoblotting to compare the gene expression levels related to the epithelial-mesenchymal phenotype in 143B-R cells and 143B-P cells. 143B-R cells showed gain of the epithelial marker, ZO-1 (P = 0.012) and loss of mesenchymal markers, vimentin (P < 0.001), Snail (P < 0.001), and Slug (P < 0.001), compared to 143B-P cells (**Figure 4**). These results indicate that EMT is, at least in part, related to the metastatic process in osteosarcoma, as well as carcinoma cells, which is consistent to previous reports (27–37, 46–48), and also suggest that the modulation of epithelial-mesenchymal phenotype may, at least in part, be related to the relationship of methionine addiction and malignancy of osteosarcoma cells.

### The status of histone-H3 lysine-methylation was significantly altered in methionine-addicted parental osteosarcoma cells and methionine-independent revertant osteosarcoma cells

**Figure 5:**
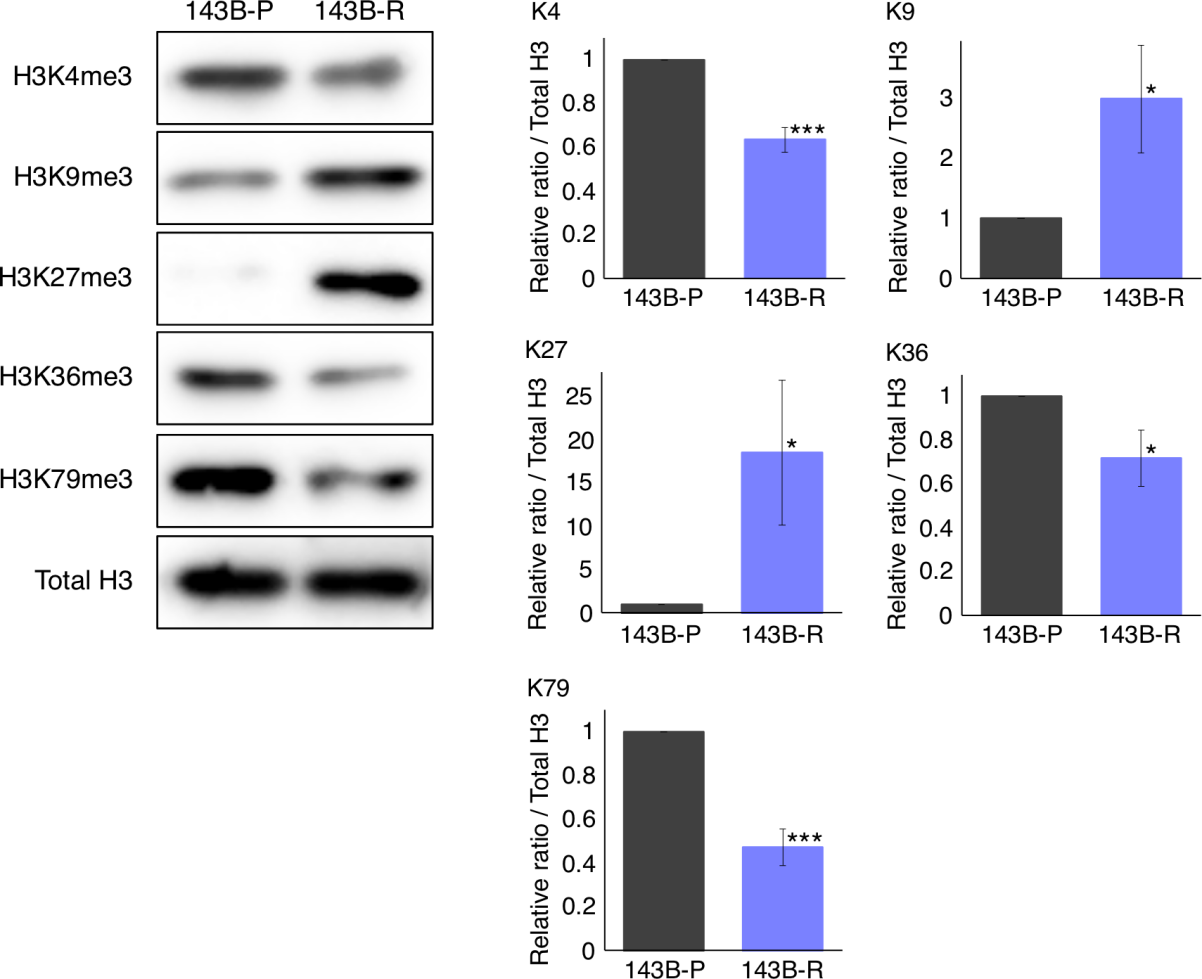
The status of the histone-H3 lysine-methylation in 143B-P and 143B-R cells, with western immunoblotting. 143B-P: methionine-addicted parental 143B osteosarcoma cells, 143B-R: methionine-independent 143B osteosarcoma cells. *P < 0.05, ***P < 0.001.

To compare the status of histone-H3 lysine-methylation in 143B-R and 143B-P, western immunoblotting was performed. The levels of histone H3K9me3 and H3K27me3 were increased (P = 0.035, P = 0.042, respectively) and the levels of histone H3K4me3, H3K36me3, and H3K79me3 were decreased (P < 0.001, P = 0.036, P < 0.001, respectively) in 143B-R cells, compared to 143B-P cells (**Figure 5**). These results suggest that unbalanced histone-H3 lysine-methylation status may be involved in the relationship of methionine addiction and malignancy of osteosarcoma cells.

## Discussion

The present study showed that revertant 143B-R cells, selected from parental 143B-P cells long-term in low-methionine media, became more resistant to methionine restriction than 143B-P cells. 143B-R cells had reduced cell migration and invasion capacity *in vitro* and reduced tumor growth and loss of metastatic potential *in vivo*, indicating they lost malignancy, which is consistent with other methionine-independent revertant cells selected from other methionine-addicted cancer types (13, 15, 16, 21, 22). These results strongly support the concept that methionine addiction is closely related to malignancy.

In the present study, histone H3K9me3 and H3K27me3 were increased, and histone H3K4me3, H3K36me3, and H3K79me3 were decreased, in 143B-R cells. Histone H3K9me3 and H3K27me3 are involved in gene repression and histone H3K4, H3K36, and H3K79 are involved in gene promotion (52–57). Although which of the histone-H3 lysine-methylation-modifications are directly involved in specific gene-modifications is still uncertain (58, 59), the results of the present study suggest that shifting the balance in histone methylases might be a way to decrease the malignant potential of cells, and the loss of malignancy of the methionine-independent revertant cells may be related to regulation of specific gene-expression changes *via* these histone-H3 lysine-mark modifications. The present study suggests that histone-H3 lysine-methylation may be involved in the relationship of malignancy and methionine addiction, as previously reported (15, 16). Although, in the present study, the western immunoblotting clearly shows the changes in histone-H3 lysine-methylation when 143B-P cells revert to 143B-R cells, future experiments will further investigate changes in histone-H3 lysine-methylation in 143B-P and 143B-R cells in IHC experiments. The mechanism of how the changes in the methylation of the histone-H3 lysine marks, including H3K4me3, H3K9me3, H3K27me3, H3K36me3, and H3K79me3, affects properties of sarcoma will be investigated in future studies.

The histone-H3 lysine-trimethylation, including H3K4me3, H3K9me3, H3K27me3, H3K36me3, and H3K79me3, were all reduced in methionine-independent revertant cells of methionine-addicted HCT116 colon cancer cells and H460 lung cancer cells (15). In the present study, selection for 143B-R cells was performed more strictly and for a longer period than in previous studies, with 4 cycles of the following selection procedure repeated: cells were cultured in methionine depleting medium until almost all the cells died, followed by rescue of any remaining live cells in normal medium and subsequent passage. Moreover, 143B osteosarcoma cells are much more sensitive to methionine restriction than HCT116 colon cancer cells and H460 lung cancer cells (IC_50_ of rMETase: 143B: 0.20 U/ml; HCT116: 0.71 U/ml; H460: 1.14 U/ml), suggesting 143B cells are more methionine-addicted than HCT116 colon-cancer cells or H460 lung-cancer cells (12). These differences might have contributed to selection of 143B-R cells with different histone-H3 lysine methylation changes from the revertant cells of HCT116 or H460 cells.

In the present study, 143B-R cells showed gain of an epithelial marker, ZO-1, and loss of mesenchymal markers, vimentin, Snail, and Slug, also contributing to the benign characteristics of 143B-R cells, which formed no spontaneous metastases in the lungs, in contrast to 143B-P cells. These results suggest that 143B-R cells have lost their metastatic potential, to which the modulation of epithelial-mesenchymal phenotype and alteration of histone-H3 lysine-methylation may be related. Changes in histone-H3 lysine-methylation, including H3K4me3, H3K9me3, H3K27me3, H3K36me3, and H3K79me3, are associated with epithelial-mesenchymal phenotype changes (60–62). Borek, almost 60 years ago, suggested unbalanced methylation is a basis of cancer (63). The present results support Borek’s hypothesis.

The present study demonstrated that reversion of methionine addiction of cancer cells to methionine-independence results in loss of malignancy, and showed the modulation of the epithelial-mesenchymal phenotype and unbalanced histone-H3 lysine-methylation status are involved in the relationship of methionine addiction and malignancy. Methionine addiction of cancer is known as the Hoffman Effect (64). Future studies will focus more on the relationship of the modulation of the epithelial-mesenchymal phenotype and histone-H3 lysine-methylation status to methionine addiction and malignancy, by upregulating or blocking the changes we have observed.

## Data availability statement

Datasets are available on request: The raw data supporting the conclusions of this article will be made available by the authors, without undue reservation.

## Ethics statement

The animal study was reviewed and approved by Institutional Animal Care and Use Committee Assurance Number A3873-1.

## Author contributions

YA, YT and RMH were involved in study conception and design. YA, QH, JY, YK, NM, KO, KH, and JW were involved in acquisition of data. YA, YT, JY, YK, NM, KO, SI, MB, SC, KN, and RMH analyzed and interpreted data. YA, YT, and RMH wrote the manuscript. All authors contributed to the article and approved the submitted version.

## Funding

This study was funded in part by the Robert M. Hoffman Foundation for Cancer Research.

## Acknowledgments

This article is dedicated to the memory of A. R. Moossa, MD, Sun Lee, MD, Professor Li Jiaxi, Masaki Kitajima, MD, Joseph R. Bertino, MD, Shigeo Yagi, PhD, and Jack Geller, MD.

## Conflict of interest

QH is an employee of AntiCancer Inc. YA, JY, YK, NM, KO, KH, and RMH are or were unsalaried associates of AntiCancer Inc.

All authors declare that the research was conducted in the absence of any commercial or financial relationships that could be construed as a potential conflict of interest.

## Publisher’s note

All claims expressed in this article are solely those of the authors and do not necessarily represent those of their affiliated organizations, or those of the publisher, the editors and the reviewers. Any product that may be evaluated in this article, or claim that may be made by its manufacturer, is not guaranteed or endorsed by the publisher.

## References

[1] Sugimura T Birnbaum SM Winitz M Greenstein JP. Quantitative nutritional studies with water-soluble, chemically defined diets. VIII. the forced feeding of diets each lacking in one essential amino acid. Arch Biochem Biophys (1959) 81(2):448–55. doi: 10.1016/0003-9861(59)90225-5 13638009

[2] Chello PL Bertino JR. Dependence of 5-methyltetrahydrofolate utilization by L5178Y murine leukemia cells *in vitro* on the presence of hydroxycobalamin and transcobalamin II. Cancer Res (1973) 33(8):1898–904. 4737200

[3] Halpern BC Clark BR Hardy DN Halpern RM Smith RA. The effect of replacement of methionine by homocystine on survival of malignant and normal adult mammalian cells in culture. Proc Natl Acad Sci U S A. (1974) 71(4):1133–6. doi: 10.1073/pnas.71.4.1133 PMC388177 4524624

[4] Hoffman RM Erbe RW. High *in vivo* rates of methionine biosynthesis in transformed human and malignant rat cells auxotrophic for methionine. Proc Natl Acad Sci U S A. (1976) 73(5):1523–7. doi: 10.1073/pnas.73.5.1523 PMC430329 179090

[5] Hoffman RM Coalson DW Jacobsen SJ Erbe RW. Folate polyglutamate and monoglutamate accumulation in normal and SV40-transformed human fibroblasts. J Cell Physiol (1981) 109(3):497–505. doi: 10.1002/jcp.1041090316 6274882

[6] Coalson DW Mecham JO Stern PH Hoffman RM. Reduced availability of endogenously synthesized methionine for s-adenosylmethionine formation in methionine-dependent cancer cells. Proc Natl Acad Sci U S A. (1982) 79(14):4248–51. doi: 10.1073/pnas.79.14.4248 PMC346647 6289297

[7] Stern PH Mecham JO Wa.llace CD Hoffman RM. Reduced free-methionine in methionine-dependent SV40-transformed human fibroblasts synthesizing apparently normal amounts of methionine. J Cell Physiol (1983) 117(1):9–14. doi: 10.1002/jcp.1041170103 6311851

[8] Stern PH Wallace CD Hoffman RM. Altered methionine metabolism occurs in all members of a set of diverse human tumor cell lines. J Cell Physiol (1984) 119(1):29–34. doi: 10.1002/jcp.1041190106 6707100

[9] Stern PH Hoffman RM. Elevated overall rates of transmethylation in cell lines from diverse human tumors. In Vitro. (1984) 20(8):663–70. doi: 10.1007/BF02619617 6500606

[10] Judde JG Ellis M Frost P. Biochemical analysis of the role of transmethylation in the methionine dependence of tumor cells. Cancer Res (1989) 49(17):4859–65. 2503245

[11] Wang Z Yip LY Lee JHJ Wu Z Chew HY Chong PKW. Methionine is a metabolic dependency of tumor-initiating cells. Nat Med (2019) 25(5):825–37. doi: 10.1038/s41591-019-0423-5 31061538

[12] Yamamoto J Han Q Inubushi S Sugisawa N Hamada K Nishino H. Histone methylation status of H3K4me3 and H3K9me3 under methionine restriction is unstable in methionine-addicted cancer cells, but stable in normal cells. Biochem Biophys Res Commun (2020) 533(4):1034–8. doi: 10.1016/j.bbrc.2020.09.108 33019978

[13] Yamamoto J Aoki Y Han Q Sugisawa N Sun YU Hamada K. Reversion from methionine addiction to methionine independence results in loss of tumorigenic potential of highly-malignant lung-cancer cells. Anticancer Res (2021) 41(2):641–3. doi: 10.21873/anticanres.14815 33517268

[14] Aoki Y Tome Y Han Q Yamamoto J Hamada K Masaki N. Histone H3 lysine-trimethylation markers are decreased by recombinant methioninase and increased by methotrexate at concentrations which inhibit methionine-addicted osteosarcoma cell proliferation. Biochem Biophys Rep (2021) 28:101177. doi: 10.1016/j.bbrep.2021.101177 34877414 PMC8633566

[15] Yamamoto J Inubushi S Han Q Tashiro Y Sugisawa N Hamada K. Linkage of methionine addiction, histone lysine hypermethylation, and malignancy. iScience (2022) 25(4):104162. doi: 10.1016/j.isci.2022.104162 35434545 PMC9010622

[16] Yamamoto J Aoki Y Inubushi S Han Q Hamada K Tashiro Y. Extent and instability of trimethylation of histone H3 lysine increases with degree of malignancy and methionine addiction. Cancer Genomics Proteomics. (2022) 19(1):12–8. doi: 10.21873/cgp.20299 PMC8717956 34949655

[17] Sedillo JC Cryns VL. Targeting the methionine addiction of cancer. Am J Cancer Res (2022) 12(5):2249–76. PMC9185618 35693095

[18] Sowers ML Sowers LC. Glioblastoma and methionine addiction. Int J Mol Sci (2022) 23(13):7156. doi: 10.3390/ijms23137156 35806160 PMC9266821

[19] Aoki Y Tome Y Han Q Yamamoto J Hamada K Masaki N. Deletion of MTAP highly sensitizes osteosarcoma cells to methionine restriction with recombinant methioninase. Cancer Genomics Proteomics. (2022) 19(3):299–304. doi: 10.21873/cgp.20321 35430564 PMC9016482

[20] Hoffman RM Jacobsen SJ Erbe RW. Reversion to methionine independence by malignant rat and SV40-transformed human fibroblasts. Biochem Biophys Res Commun (1978) 82(1):228–34. doi: 10.1016/0006-291x(78)90600-9 208554

[21] Hoffman RM Jacobsen SJ Erbe RW. Reversion to methionine independence in simian virus 40-transformed human and malignant rat fibroblasts is associated with altered ploidy and altered properties of transformation. Proc Natl Acad Sci U S A. (1979) 76(3):1313–7. doi: 10.1073/pnas.76.3.1313 PMC383241 220612

[22] Borrego SL Fahrmann J Datta R Stringari C Grapov D Zeller M. Metabolic changes associated with methionine stress sensitivity in MDA-MB-468 breast cancer cells. Cancer Metab (2016) 4:9. doi: 10.1186/s40170-016-0148-6 27141305 PMC4852440

[23] Aoki Y Yamamoto J Tome Y Hamada K Masaki N Inubushi S. Over-methylation of histone H3 lysines is a common molecular change among the three major types of soft-tissue sarcoma in patient-derived xenograft (PDX) mouse models. Cancer Genomics Proteomics (2021) 18(6):715–21. doi: 10.21873/cgp.20292 PMC8569816 34697064

[24] Tan Y Xu M Tan X Wang X Saikawa Y Nagahama T. Overexpression and large-scale production of recombinant l-methionine-alpha-deamino-gamma-mercaptomethane-lyase for novel anticancer therapy. Protein Expr Purif. (1997) 9(2):233–45. doi: 10.1006/prep.1996.0700 9056489

[25] Saraf AJ Fenger JM Roberts RD. Osteosarcoma: Accelerating progress makes for a hopeful future. Front Oncol (2018) 8:4. doi: 10.3389/fonc.2018.00004 29435436 PMC5790793

[26] Kalluri R Weinberg RA. The basics of epithelial-mesenchymal transition. J Clin Invest. (2009) 119(6):1420–8. doi: 10.1172/JCI39104 PMC2689101 19487818

[27] Guo Y Zi X Koontz Z Kim A Xie J Gorlick R. Blocking Wnt/LRP5 signaling by a soluble receptor modulates the epithelial to mesenchymal transition and suppresses met and metalloproteinases in osteosarcoma saos-2 cells. J Orthop Res (2007) 25(7):964–71. doi: 10.1002/jor.20356 17318900

[28] Hou CH Lin FL Hou SM Liu JF. Cyr61 promotes epithelial-mesenchymal transition and tumor metastasis of osteosarcoma by raf-1/MEK/ERK/Elk-1/TWIST-1 signaling pathway. Mol Cancer. (2014) 13:236. doi: 10.1186/1476-4598-13-236 25326651 PMC4210521

[29] Meng Q Ren C Wang L Zhao Y Wang S. Knockdown of ST6Gal-I inhibits the growth and invasion of osteosarcoma MG-63 cells. BioMed Pharmacother. (2015) 72:172–8. doi: 10.1016/j.biopha.2015.04.020 26054692

[30] Feng ZM Guo SM. Tim-3 facilitates osteosarcoma proliferation and metastasis through the NF-κB pathway and epithelial-mesenchymal transition. Genet Mol Res (2016) 15(3):1-9. doi: 10.4238/gmr.15037844 27706678

[31] Liu W Qiao RH Wang DM Huang XW Li B Wang D. UHRF1 promotes human osteosarcoma cell invasion by downregulating the expression of e−cadherin in an Rb1−dependent manner. Mol Med Rep (2016) 13(1):315–20. doi: 10.3892/mmr.2015.4515 26548607

[32] Lv YF Dai H Yan GN Meng G Zhang X Guo QN. Downregulation of tumor suppressing STF cDNA 3 promotes epithelial-mesenchymal transition and tumor metastasis of osteosarcoma by the Wnt/GSK-3β/β-catenin/Snail signaling pathway. Cancer Lett (2016) 373(2):164–73. doi: 10.1016/j.canlet.2016.01.046 26845447

[33] Chen J Yan D Wu W Zhu J Ye W Shu Q. MicroRNA-130a promotes the metastasis and epithelial-mesenchymal transition of osteosarcoma by targeting PTEN. Oncol Rep (2016) 35(6):3285–92. doi: 10.3892/or.2016.4719 27035216

[34] Somarelli JA Shetler S Jolly MK Wang X Bartholf Dewitt S Hish AJ. Mesenchymal-epithelial transition in sarcomas is controlled by the combinatorial expression of MicroRNA 200s and GRHL2. Mol Cell Biol (2016) 36(19):2503–13. doi: 10.1128/MCB.00373-16 PMC5021378 27402864

[35] Chen Y Zhang K Li Y He Q. Estrogen-related receptor α participates transforming growth factor-β (TGF-β) induced epithelial-mesenchymal transition of osteosarcoma cells. Cell Adh Migr. (2017) 11(4):338–46. doi: 10.1080/19336918.2016.1221567 PMC5569972 27532429

[36] Tian H Zhou T Chen H Li C Jiang Z Lao L. Bone morphogenetic protein-2 promotes osteosarcoma growth by promoting epithelial-mesenchymal transition (EMT) through the wnt/β-catenin signaling pathway. J Orthop Res (2019) 37(7):1638–48. doi: 10.1002/jor.24244 30737824

[37] Wang S Zhao G Zhao S Qiao Y Yang H. The effects of interleukin-33 (IL-33) on osteosarcoma cell viability, apoptosis, and epithelial-mesenchymal transition are mediated through the PI3K/AKT pathway. Med Sci Monit (2020) 26:e920766. doi: 10.12659/MSM.920766 32312946 PMC7191962

[38] Saito T Nagai M Ladanyi M. SYT-SSX1 and SYT-SSX2 interfere with repression of e-cadherin by snail and slug: A potential mechanism for aberrant mesenchymal to epithelial transition in human synovial sarcoma. Cancer Res (2006) 66(14):6919–27. doi: 10.1158/0008-5472.CAN-05-3697 16849535

[39] Fitzgerald MP Gourronc F Teoh ML Provenzano MJ Case AJ Martin JA. Human chondrosarcoma cells acquire an epithelial-like gene expression pattern *via* an epigenetic switch: Evidence for mesenchymal-epithelial transition during sarcomagenesis. Sarcoma (2011) 2011:598218. doi: 10.1155/2011/598218 21559267 PMC3087947

[40] Qi Y Wang CC He YL Zou H Liu CX Pang LJ. The correlation between morphology and the expression of TGF-β signaling pathway proteins and epithelial-mesenchymal transition-related proteins in synovial sarcomas. Int J Clin Exp Pathol (2013) 6(12):2787–99. PMC3843259 24294365

[41] Lee KW Lee NK Ham S Roh TY Kim SH. Twist1 is essential in maintaining mesenchymal state and tumor-initiating properties in synovial sarcoma. Cancer Lett (2014) 343(1):62–73. doi: 10.1016/j.canlet.2013.09.013 24051309

[42] Srivastava RK Kaylani SZ Edrees N Li C Talwelkar SS Xu J. GLI inhibitor GANT-61 diminishes embryonal and alveolar rhabdomyosarcoma growth by inhibiting Shh/AKT-mTOR axis. Oncotarget (2014) 5(23):12151–65. doi: 10.18632/oncotarget.2569 PMC4322980 25432075

[43] Li G Yang Y Xu S Ma L He M Zhang Z. Slug signaling is up-regulated by CCL21/CCR7 [corrected] to induce EMT in human chondrosarcoma. Med Oncol (2015) 32(2):478. doi: 10.1007/s12032-014-0478-6 25556164

[44] Yang P Wang G Huo H Li Q Zhao Y Liu Y. SDF-1/CXCR4 signaling up-regulates survivin to regulate human sacral chondrosarcoma cell cycle and epithelial-mesenchymal transition *via* ERK and PI3K/AKT pathway. Med Oncol (2015) 32(1):377. doi: 10.1007/s12032-014-0377-x 25428386

[45] Skrzypek K Kot M Konieczny P Nieszporek A Kusienicka A Lasota M. SNAIL promotes metastatic behavior of rhabdomyosarcoma by increasing EZRIN and AKT expression and regulating MicroRNA networks. Cancers (Basel). (2020) 12(7):1870. doi: 10.3390/cancers12071870 PMC7408994 32664538

[46] Sannino G Marchetto A Kirchner T Grünewald TGP. Epithelial-to-Mesenchymal and mesenchymal-to-Epithelial transition in mesenchymal tumors: A paradox in sarcomas? Cancer Res (2017) 77(17):4556–61. doi: 10.1158/0008-5472.CAN-17-0032 28811330

[47] Yu X Yustein JT Xu J. Research models and mesenchymal/epithelial plasticity of osteosarcoma. Cell Biosci (2021) 11(1):94. doi: 10.1186/s13578-021-00600-w 34022967 PMC8141200

[48] Damerell V Pepper MS Prince S. Molecular mechanisms underpinning sarcomas and implications for current and future therapy. Signal Transduct Target Ther (2021) 6(1):246. doi: 10.1038/s41392-021-00647-8 34188019 PMC8241855

[49] Aoki Y Tome Y Wu NF Yamamoto J Hamada K Han Q. Oral-recombinant methioninase converts an osteosarcoma from docetaxel-resistant to -sensitive in a clinically-relevant patient-derived orthotopic-xenograft (PDOX) mouse model. Anticancer Res (2021) 41(4):1745–51. doi: 10.21873/anticanres.14939 33813378

[50] Aoki Y Tome Y Han Q Yamamoto J Hamada K Masaki N. Oral-recombinant methioninase converts an osteosarcoma from methotrexate-resistant to -sensitive in a patient-derived orthotopic-xenograft (PDOX) mouse model. Anticancer Res (2022) 42(2):731–7. doi: 10.21873/anticanres.15531 35093871

[51] Booher K Lin DW Borrego SL Kaiser P. Downregulation of Cdc6 and pre-replication complexes in response to methionine stress in breast cancer cells. Cell Cycle (2012) 11(23):4414–23. doi: 10.4161/cc.22767 PMC3552924 23159852

[52] Briggs SD Xiao T Sun ZW Caldwell JA Shabanowitz J Hunt DF. Gene silencing: Trans-histone regulatory pathway in chromatin. Nature (2002) 418(6897):498. doi: 10.1038/nature00970 12152067

[53] Bannister AJ Schneider R Kouzarides T. Histone methylation: dynamic or static? Cell (2002) 109(7):801–6. doi: 10.1016/s0092-8674(02)00798-5 12110177

[54] Bannister AJ Kouzarides T. Reversing histone methylation. Nature (2005) 436(7054):1103–6. doi: 10.1038/nature04048 16121170

[55] Kouzarides T. Chromatin modifications and their function. Cell (2007) 128(4):693–705. doi: 10.1016/j.cell.2007.02.005 17320507

[56] Cedar H Bergman Y. Linking DNA methylation and histone modification: Patterns and paradigms. Nat Rev Genet (2009) 10(5):295–304. doi: 10.1038/nrg2540 19308066

[57] Greer EL Shi Y. Histone methylation: A dynamic mark in health, disease and inheritance. Nat Rev Genet (2012) 13(5):343–57. doi: 10.1038/nrg3173 PMC4073795 22473383

[58] Henikoff S Shilatifard A. Histone modification: Cause or cog? Trends Genet (2011) 27(10):389–96. doi: 10.1016/j.tig.2011.06.006 21764166

[59] Michalak EM Burr ML Bannister AJ Dawson MA. The roles of DNA, RNA and histone methylation in ageing and cancer. Nat Rev Mol Cell Biol (2019) 20(10):573–89. doi: 10.1038/s41580-019-0143-1 31270442

[60] McDonald OG Wu H Timp W Doi A Feinberg AP. Genome-scale epigenetic reprogramming during epithelial-to-mesenchymal transition. Nat Struct Mol Biol (2011) 18(8):867–74. doi: 10.1038/nsmb.2084 PMC3150339 21725293

[61] Wang Y Shang Y. Epigenetic control of epithelial-to-mesenchymal transition and cancer metastasis. Exp Cell Res (2013) 319(2):160–9. doi: 10.1016/j.yexcr.2012.07.019 22935683

[62] Sun L Fang J. Epigenetic regulation of epithelial-mesenchymal transition. Cell Mol Life Sci (2016) 73(23):4493–515. doi: 10.1007/s00018-016-2303-1 PMC5459373 27392607

[63] Borek E Srinivasan PR. The methylation of nucleic acids. Annu Rev Biochem (1966) 35(1):275–98. doi: 10.1146/annurev.bi.35.070166.001423

[64] Kaiser P. Methionine dependence of cancer. Biomolecules (2020) 10(4):568. doi: 10.3390/biom10040568 PMC7226524 32276408

